# Establishment of a 3D co‐culture model to investigate the role of primary fibroblasts in ductal carcinoma in situ of the breast

**DOI:** 10.1002/cnr2.1771

**Published:** 2022-12-19

**Authors:** Marina Sourouni, Carl Opitz, Isabel Radke, Ludwig Kiesel, Joke Tio, Martin Götte, Marie‐Kristin von Wahlde

**Affiliations:** ^1^ Department of Obstetrics and Gynecology, Breast Center University Hospital Münster Münster Germany

**Keywords:** 3D co‐culture, ductal carcinoma in situ, invasion, tumor microenvironment

## Abstract

**Background:**

Ductal carcinoma in situ (DCIS) is a precursor form of breast cancer. 13%–50% of these lesions will progress to invasive breast cancer, but the individual progression risk cannot be estimated. Therefore, all patients receive the same therapy, resulting in potential overtreatment of a large proportion of patients.

**Aims:**

The role of the tumor microenvironment (TME) and especially of fibroblasts appears to be critical in DCIS development and a better understanding of their role may aid individualized treatment.

**Methods and results:**

Primary fibroblasts isolated from benign or malignant punch biopsies of the breast and MCF10DCIS.com cells were seeded in a 3D cell culture system. The fibroblasts were cultured in a type I collagen layer beneath a Matrigel layer with MCF10DCIS.com cells. Dye‐quenched (DQ) fluorescent collagen I and IV were used in collagen and Matrigel layer respectively to demonstrate proteolysis. Confocal microscopy was performed on day 2, 7, and 14 to reveal morphological changes, which could indicate the transition to an invasive phenotype.

MCF10DCIS.com cells form smooth, round spheroids in co‐culture with non‐cancer associated fibroblasts (NAFs). Spheroids in co‐culture with tumor‐associated fibroblasts (TAFs) appear irregularly shaped and with an uneven surface; similar to spheroids formed from invasive cells. Therefore, these morphological changes represent the progression of an in situ to an invasive phenotype. In addition, TAFs show a higher proteolytic activity compared to NAFs. The distance between DCIS cells and fibroblasts decreases over time.

**Conclusion:**

The TAFs seem to play an important role in the progression of DCIS to invasive breast cancer. The better characterization of the TME could lead to the identification of DCIS lesions with high or low risk of progression. This could enable personalized oncological therapy, prevention of overtreatment and individualized hormone replacement therapy after DCIS.

## 1 INTRODUCTION

Breast cancer is the most common cancer type in women divided into an invasive and non‐invasive histological type(s) according to the WHO classification.^1^ The risk of progression to an invasive form varies among pre‐invasive lesions. The highest risk for transition to an invasive breast cancer and thus to a potentially life‐threatening disease is seen in ductal carcinoma in situ (DCIS) with 13%–50% of the cases.^2^ So far, it is not possible to predict which DCIS lesion will become invasive and therefore all patients with a DCIS are treated equally resulting in over‐treatment of some patients.

Therapy of DCIS includes surgical treatment either breast‐conserving or a mastectomy depending on the tumor‐size and breast relation. The possibility of radiotherapy after breast‐conserving surgery and/or endocrine therapy should be discussed with the patient individually based on a risk–benefit assessment, considering potential adverse effects.^3^, ^4^, ^5^

Possible therapy associated morbidity through radiation or surgery can affect the lifestyle of the patient and reduce the quality of life.^6^ Therefore, DCIS over‐treatment leads to potential adverse effects regarding patients' health and constitutes a burden on the health system.^7^ Moreover, there is no data regarding the use of hormone replacement therapy after DCIS, which is usually avoided due to the fear this could trigger an invasive transformation/reformation.^8^ Prognostic parameters are needed to assess the patient's individual risk. In case of a low risk, interventional therapy could be replaced by regular follow up.^9^

DCIS is considered a non‐obligatory precursor to invasive breast cancer^10^ and there are two progression theories; the ‘genetic’ and the ‘non‐genetic’ theory. The genetic theory is supported by studies that have revealed the genetic similarity and the likely common origin of DCIS and invasive carcinoma.^11^, ^12^, ^13^ Clinical observations have also shown that the two entities are often located at the same anatomical site or directly next to each other, which suggests an evolutionary continuum. Gene expression analysis has been conducted and provides evidence of a common genetic background.^14^ Other studies have shown a significantly different expression pattern for distinct genes, which could indicate driver pathways that play an important role in the progression from DCIS to invasive breast cancer.^15^, ^16^, ^17^ However, a single mutation as a cause for the progression seems unlikely. So far, no biomarker could be found to clinically predict the progression from DCIS to invasive breast cancer.

The non‐genetic theory suggests that the tumor microenvironment (TME) plays a decisive role in the progression of DCIS to invasive breast cancer.^18^ DCIS is surrounded by an outer layer of myoepithelial cells and an intact basement membrane. The layer of normal myoepithelial cells acts as a “gatekeeper” and has tumor suppressive effects on the in situ lesion.^19^ Various studies have shown that the loss of this suppressive effect leads to the progression to invasive breast cancer.^19^, ^20^ Except from a physical barrier against invasion, myoepithelial cells also secrete various extracellular matrix components (ECM) which inhibit the invasive capacity of DCIS in a paracrine way.^21^ Increased stromal cell expression of ECM‐modifying enzymes^17^, ^22^ as well as glucocorticoids seem to favor the progression of DCIS to invasive breast cancer in vitro and in vivo.^23^ Stromal fibroblasts as well as tumor infiltrating lymphocytes (TILs) and their T‐ to B‐cell ratio also seem to play a role in the progression of DCIS.^17^, ^24^

Altogether, the current literature leads to the conclusion that the transition from DCIS to invasive breast cancer is largely not dependent on intrinsic mutations. The invasive potential appears to be rather due to extrinsic factors or the TME.^25^ These include the ECM, the myoepithelial cell layer, immune cells and fibroblasts. Therefore, there is a need to establish basic novel experimental models of DCIS that take the TME into account. In this study, we describe a novel 3D co‐culture model of DCIS and primary fibroblasts as a major component of the TME that can be used to study the progression of DCIS in a convenient laboratory setting. This work primarily focuses on morphological changes potentially representing an invasive phenotype in this model.

## 2 MATERIALS AND METHODS

### 2.1 Materials

All supplies and chemicals were from Sigma‐Aldrich Chemie GmbH (Taufkirchen, Germany) unless otherwise stated.

### 2.2 Cell culture

The human DCIS model cell line MCF10DCIS.com, cloned from a cell culture initiated from a xenograft lesion^26^ was obtained from Karmanos Cancer Institute (Detroit, MI, USA) and cultured in DMEM/F12 containing 20% Calf Serum und 10% Autoclaved DMSO. The human triple‐negative breast cancer cell line MDA‐MB‐231 was obtained from ATCC/LGC Promochem (Wesel, Germany) and maintained in DMEM containing 1% glutamine, 10% fetal bovine serum, and 1% penicillin/streptomycin in a humidified atmosphere of 5% CO_2_ at 37°C. Primary fibroblasts were isolated from malignant or benign punch biopsies of the breast. The study was carried out following the Declaration of Helsinki and approved by the local ethics commission (Ethikkommission der Ärztekammer Westfalen‐Lippe und der Medizinischen Fakultät der WWU; approval no.1 IX Greb 1 from 19 September 2001, updated 2012). The participants gave written informed consent. Tumor associated fibroblasts (TAFs) used derived from a moderately differentiated breast cancer (NST), ER 95%, PR 80%, Her2 neu neg, Ki 67 20% of a 62 year old patient (type 1) or from a highly differentiated mucinous breast cancer, ER 90%, PR 90%, Her2 neu neg, Ki 67 5% of a 82 year old patient (type 2). The non‐TAFs were derived either from breast tissue of a 50 year old patient with microcalcifications but no indication of malignancy or from a fibroadenoma of a 19 year old patient. The two types of non‐TAFs were used interchangeably to enable a time efficient conduction of the experiment despite their slow growth in culture. Fibroblasts were cultured in RPMI Medium containing 10% fetal bovine serum and 1% penicillin/streptomycin in a humidified atmosphere of 7% CO_2_ at 37°C .

### 2.3 3D co‐culture model

The co‐culture of DCIS cells and primary fibroblasts was based on the MAME model (mammary architecture and microenvironment engineering) of Sameni et al. and was modified to meet the needs of primary cells.^27^ Primary fibroblasts were isolated from the punch biopsies and were then cultured in a Type I collagen layer (Discovery Labware, Inc. by Corning, Bedford, MA, USA) under a Matrigel layer (Discovery Labware, Inc. by Corning, Bedford, MA, USA) with MCF10DCIS.com cells in a 6 well plate using the culture media described above. Dye‐ quenched fluorescent collagen (Invitrogen by Thermo Fisher Scientific, Eugene, USA) was used in both layers to demonstrate proteolysis (DQ‐collagen I in collagen I and DQ‐collagen IV in Matrigel). The 3D co‐culture was then examined under the confocal microscope after 2, 7, and 14 days to reveal morphological changes that could indicate the transition to an invasive phenotype. DCIS cells and fibroblasts were marked with cell tracker red, whereas degraded DQ‐collagen exhibited green fluorescence. The control with only DCIS was compared to DCIS in co‐culture with either TAFs or NAFs.

**FIGURE 1:**
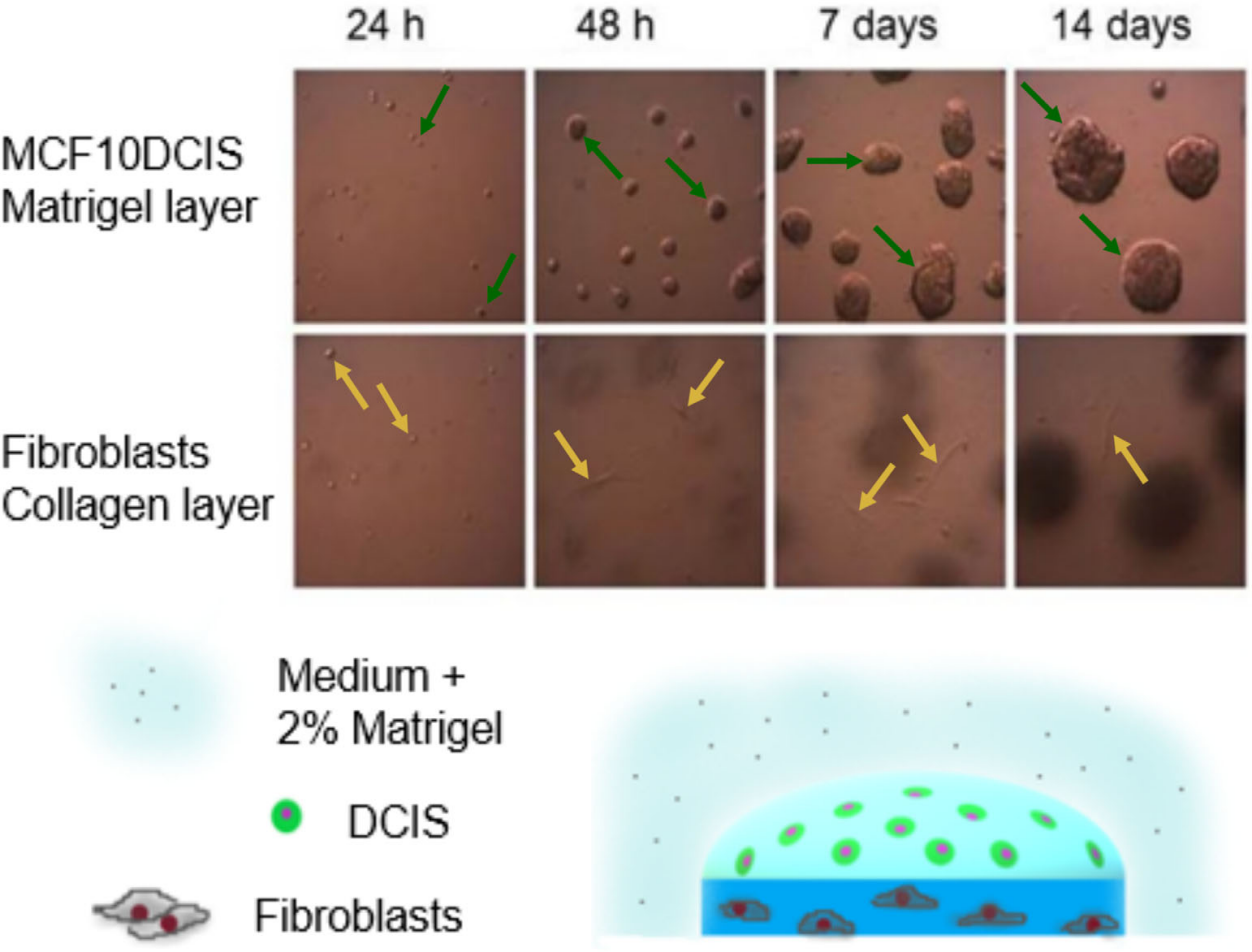
Composition of the 3D co‐culture system and morphology of MCF10A.DCIS.com cells in Matrigel and of fibroblasts in collagen after 24 h, 48 h, 7 days and 14 days. Green arrows point to typical examples of ductal carcinoma in situ (DCIS) cells forming spheroids, which grow over time. Yellow arrows point to typical examples of fibroblasts in the collagen layer. Phase contrast microscopy. Magnification = 10×‐fold

The co‐culture consists of two dye‐quenched (DQ) substrates and three layers (Figure 1). For the preparation of the reconstituted basement membrane (rBM) substrate, Matrigel (Discovery Labware, Inc. by Corning, Bedford, MA, USA) was thawed the day before at 4°C and then diluted dye‐quenched (DQ) collagen IV (Invitrogen by Thermo Fisher Scientific, Eugene, USA) in a pre‐cooled tube was added to a final concentration of 25 μg/mL. The collagen I solution was prepared on ice. Collagen I (Discovery Labware, Inc. by Corning, Bedford, MA, USA) was mixed with PBS in a ratio of 1:8. The pH was adjusted to 7.2–7.6 with 1 part of 0.16 M NaOH and checked with pH strips. DQ‐collagen I (Invitrogen by Thermo Fisher Scientific, Eugene, USA) was added to a final concentration of 25 μg/mL. The desired number of fibroblasts (10^3^) in 10 μl medium was added to 60 μl of collagen I and DQ‐collagen I mixture and constituted the bottom layer of the co‐culture The culture dish was then incubated to solidify for 30 min without CO_2_ at 37°C, followed by 10 min with 5% CO_2_ at 37°C and then brought to room temperature. On top of that, a layer of 60 μl Matrigel mixed with DQ‐collagen IV was placed. The culture dish was then incubated for 10 min at 37°C and 5% CO_2_ to solidify. A top layer of 50 μl cell suspension including the desired number of MCF10DCIS.com cells (5 × 10^3^) with 2% Matrigel was added and the culture dish was incubated for 40–60 min with 5% CO_2_ at 37°C to allow the cells to adhere. The co‐culture was then covered with 2 ml of medium with 2% Matrigel and incubated for the desired time of 14 days at 37°C and 5% CO_2_. The medium containing 1% Matrigel was changed every 3–4 days.

The two types of DQ‐collagen, fluorescently labeled collagen that generates fluorescent fragments upon proteolytic degradation, were used to display proteolysis. Optical sections were captured, processed, and reconstructed in 3D. The experiments were performed in triplicates with similar experimental conditions (plate, cells number, medium, exposure duration, etc.).

### 2.4 Light microscopy

The co‐culture was first visualized on day 2, 4, 7, and 14 under the light microscope (Axioscope Carl Zeiss Microscopy GmbH, Jena, Germany) to reveal possible morphological changes between the different wells.

### 2.5 Confocal microscopy and data analysis

The co‐culture was visualized on day 2, 7, and 14 under the confocal microscope (LSM 880 Carl Zeiss Microscopy GmbH, Jena, Germany) using two channels. The HeNe543 laser has an emission wavelength of 543 nm, thus stimulating the Celltracker fluorescent dye (541/565 nm) and enabling visualization of colored cells. The argon diode 405‐30 with an emission wavelength of 488 nm catches the DQ‐collagen signal. The power of HeNe543 laser has been set to 10% and of the argon diode 405‐30 to 20%. The pinhole was set to 1 A.U. (airy unit) so that the signal‐to‐noise ratio was as low as possible. All images were taken with an identical setting and reconstructed and processed in 3D using the Zen 2 black imaging software (Carl Zeiss Microscopy GmbH, Jena, Germany). In order to be able to present the possible morphological differences between the spheroids in the control and co‐culture, consecutive images were taken every 8 μm. Acoordingly, a measuring distance of 3.5 μm between consecutive images was selected for the significantly smaller fibroblasts, so that even small differences could be detected. For the measurement of the distance between fibroblasts and MCF10DCIS.com cells, images were taken every 15 μm.

### 2.6 Statistical analysis

For statistical analysis a two tailed t – test was performed. The significance level was set equal to 0.05.

## 3 RESULTS

### 3.1 Establishment of a DCIS – fibroblast co‐culture model

To establish a co‐culture model of MCF10DCIS.com cells and fibroblasts, primary non‐TAFs and two types of TAFs (types 1 and 2, see ‘methods’ session) were isolated. Approximately 14 days after isolation, sufficient actively proliferating fibroblasts were available for 3D co‐culture experiments.

Over the course of 14 days the DCIS cells proliferate and coalesce into grouped round structures forming spheroids, which in co‐culture with TAFs show an irregular surface structure. Fluorescent proteolytic fragments of the collagen are found in association with the surface of DCIS spheroids and of the fibroblasts.

Immediately after seeding, both cell types show a round morphology when observed by phase contrast light microscopy, while after 48 h they appeared morphologically different. The fibroblasts in the lower layer were elongated and individually located, while the MCF10DCIS.com cells formed spheroids (Figure 1). Over time these spheroids grow bigger. There was no difference in the size and/or number of formed spheroids between the different wells. Specifically, no statistically significant difference was shown in the mean diameter between control spheroids and the ones in co‐culture with NAFs (day 7: 9.26 A.U. vs. 9.36 A.U./ *p* = .85, day 14: 11.53 A.U. vs. 11.76 A.U./ *p* = .81) or the ones in co‐culture with TAFs (day 7: 9.26 A.U. vs. 9.2 A.U./ *p* = .94, day 14:11.52 A.U. vs. 11.19 A.U/ *p* = .71). There was also no statistically significant difference in the mean diameter when directly comparing spheroids formed in co‐culture with NAFs and spheroids formed in co‐culture with TAFs (day 7: 9.36 A.U. vs. 9.26 A.U./ *p* = .81, day 14: 11.76 A.U. vs 11.19 A.U./ *p* = .58).

### 3.2 Evaluation of proteolysis and cell morphology of DCIS cells by confocal microscopy

**FIGURE 2:**
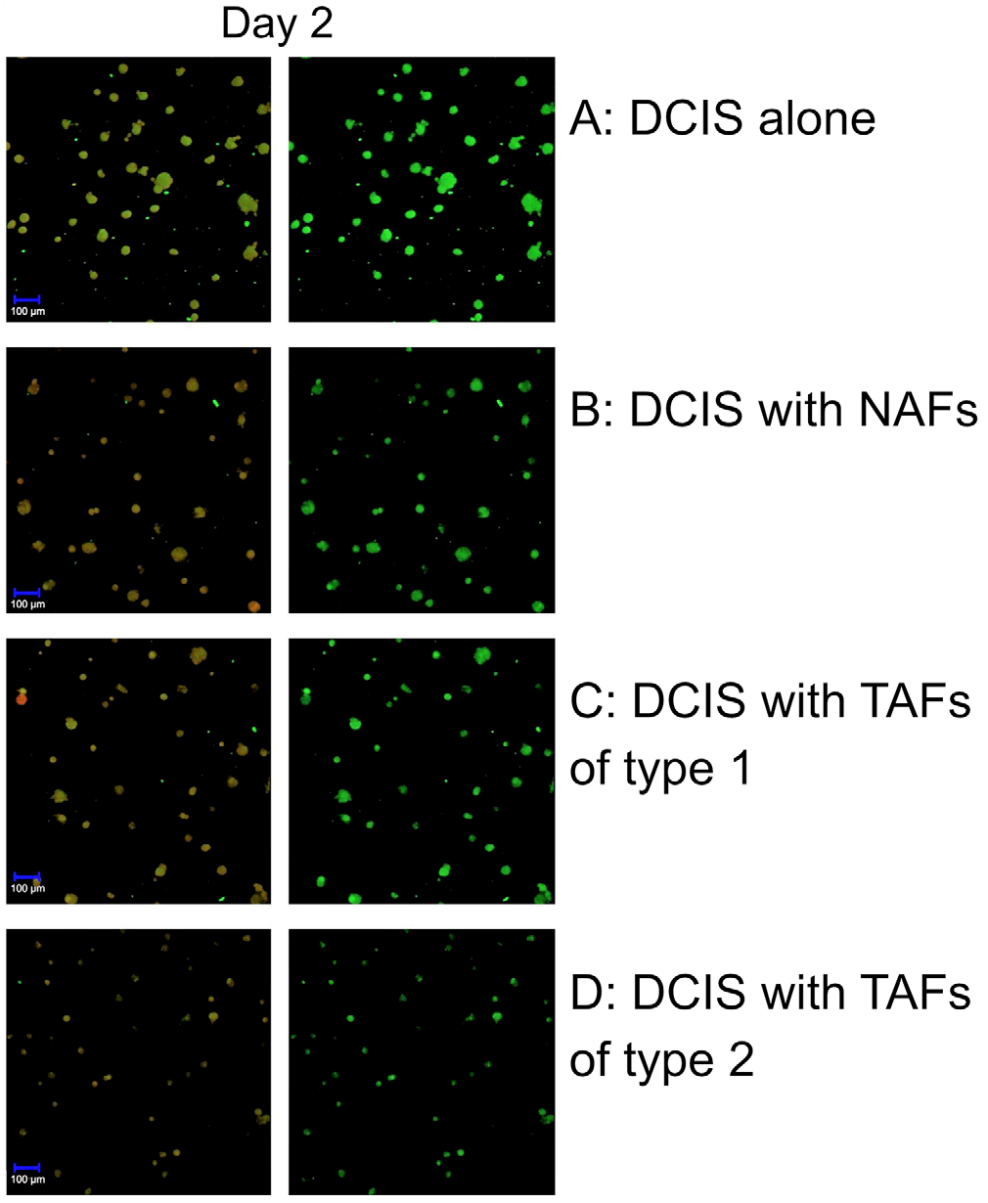
Evaluation of the 3D co‐culture system by confocal microscopy. Spheroids on day 2 with ductal carcinoma in situ (DCIS) control (A), DCIS with non‐tumor‐associated fibroblasts (NAFs) (B) and with tumor‐associated fibroblasts (TAFs) of type 1 (C) or type 2 (D). The left panels show the formation of the spheroids after labeling the DCIS cells with cell tracker red using the two channels (HeNe543, argon diode 405‐30), while the right panels show the green signal with the argon diode 405‐30 only caused by degradation of DQ‐collagen IV. Proteolysis takes place in all wells. At this time point there are no differences in the morphology of the spheroids.

**FIGURE 3:**
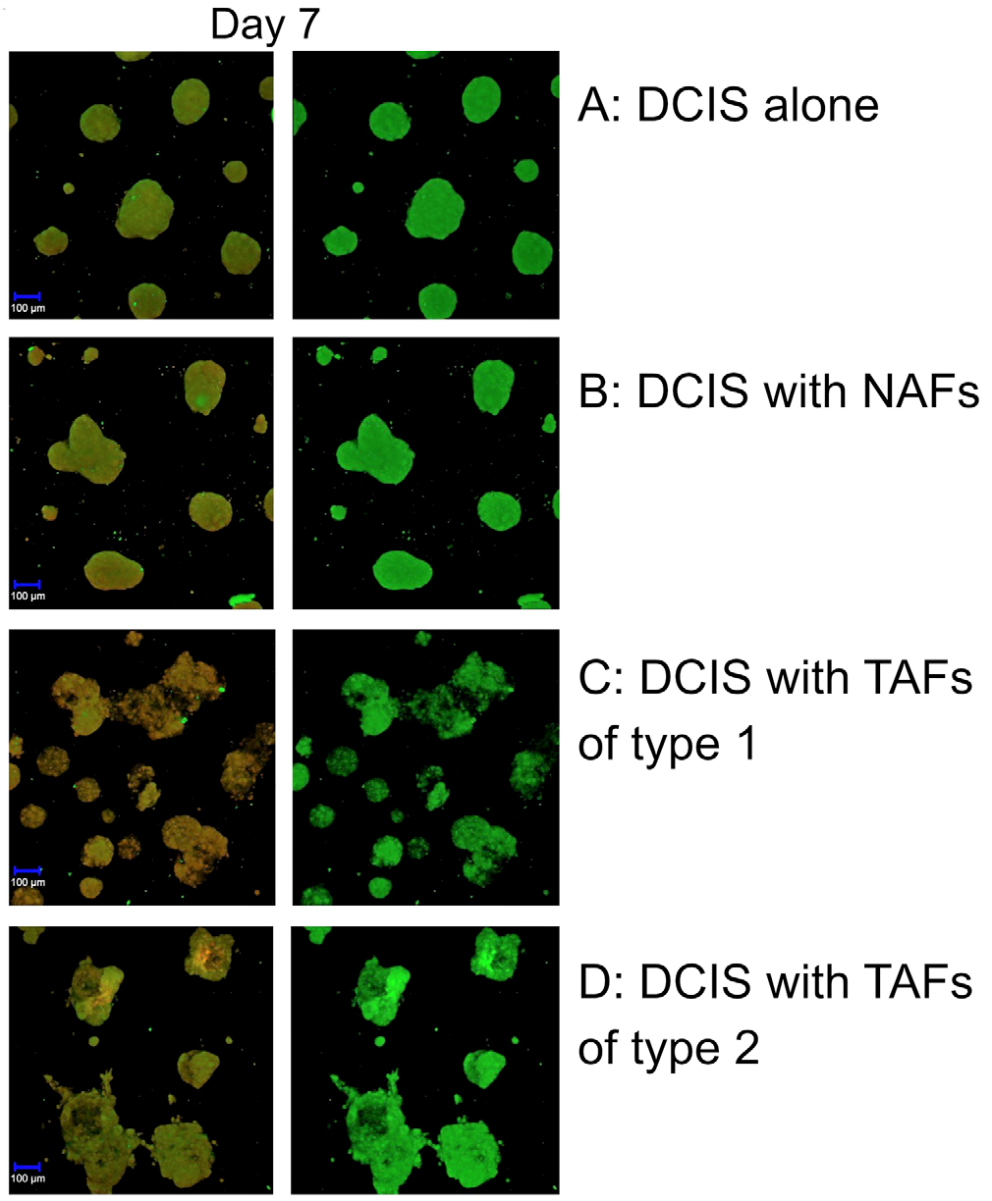
Formation of an invasive phenotype in ductal carcinoma in situ‐tumor‐associated fibroblasts (DCIS‐TAF) co‐culture. Spheroids on day 7 with DCIS control (A), DCIS with non‐tumor‐associated fibroblasts (NAFs) (B) and with tumor‐associated fibroblasts (TAFs) of type 1 (C) or type 2 (D). Spheroids in co‐culture with TAFs of type 1 (C) or 2 (D) have an uneven surface compared to the control (A) and to spheroids in co‐culture with NAFs (B). The left panels show the formation of the spheroids using the two channels (HeNe543, argon diode 405‐30), while the right panels show the green signal with the argon diode 405‐30 only caused by degradation of DQ‐collagen IV

**FIGURE 4:**
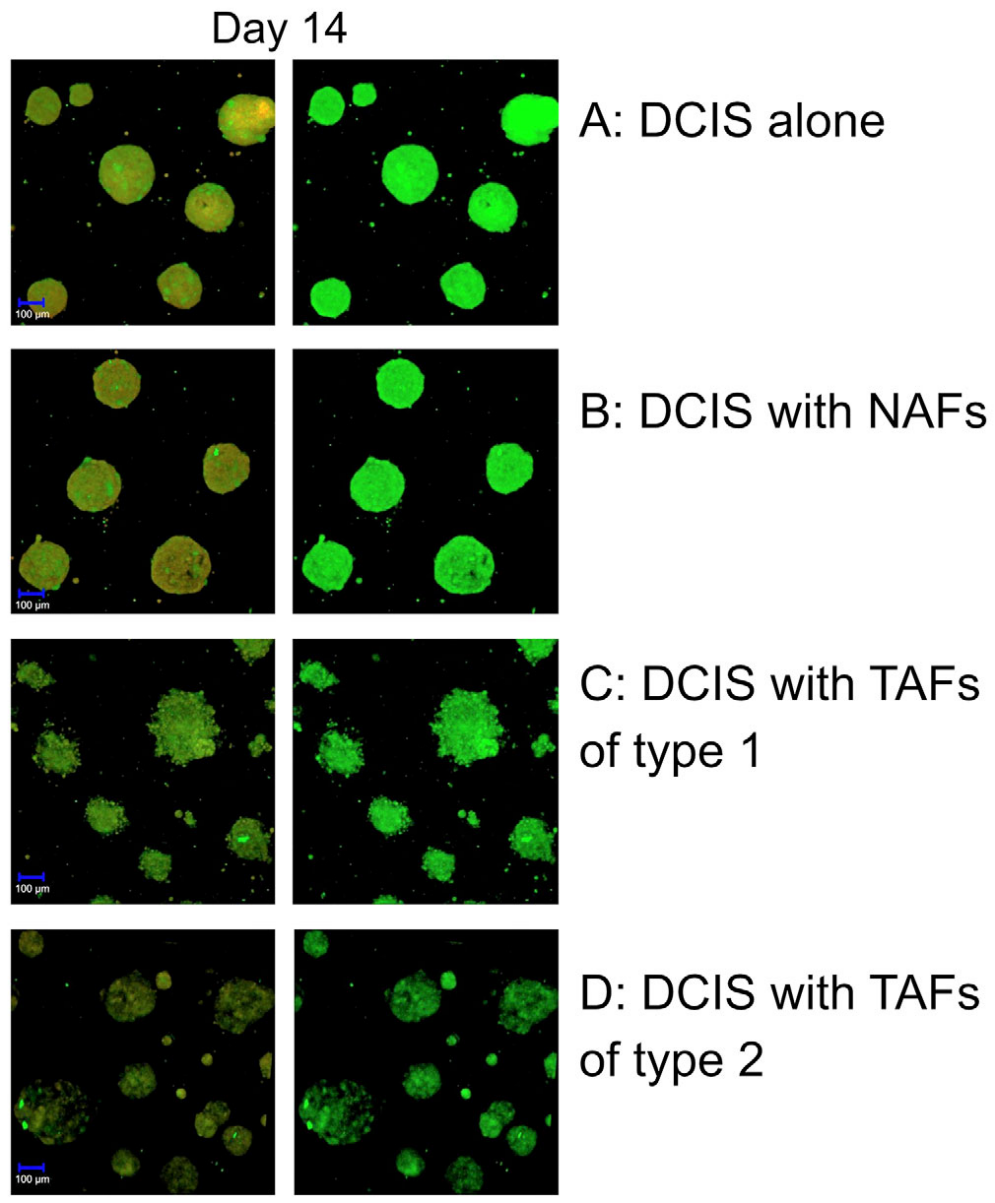
Formation of invasive phenotype in ductal carcinoma in situ‐tumor‐associated fibroblasts (DCIS‐TAF) co‐culture. Spheroids on day 14 with DCIS alone (A), DCIS with non‐tumor‐associated fibroblasts (NAFS) (B) and with tumor‐associated fibroblasts (TAFs) of type 1(C) or type 2 (D). Spheroids in co‐culture with TAFs of type 1 (C) or 2 (D) have an uneven surface compared to the control (A) and to spheroids in co‐culture with NAFs (B). The left panels show the formation of the spheroids using the two channels (HeNe543, argon diode 405‐30), while the right panels show the green signal with the argon diode 405‐30 only caused by degradation of DQ‐collagen IV

Next, we studied the morphology and proteolytic activity of cell tracker‐labeled MCF10DCIS.com cells and fibroblasts with confocal fluorescence microscopy. On day 2 of the 3D‐culture the spheroids looked small, smooth‐edged and round. Proteolysis was already ongoing during the formation of the spheroids, as indicated by the green signal caused by the degradation of DQ‐collagen IV (Figure 2). At this point, there were no morphological differences regardless of the type of TAFs (Figure 2). On day 7, the control spheroids remained smooth and round. Otherwise, the spheroids that were co‐cultured with TAFs of type 1 or 2 had an uneven appearance with the cells leaving the formation of the spheroid (Figure 3). The spheroids co‐cultured with NAFs had a morphology similar to the spheroids in the control. Spheroids retained their acquired morphology over time, so that on day 14 the spheroids in co‐culture with TAFs of type 1 or 2 appeared further irregularly shaped compared to the spheroids co‐cultured with NAFs (Figure 4). Spheroids in co‐culture with NAFs showed the same phenotype as the control spheroids. The proteolysis that has taken place is further displayed by the green fluorescence of the DQ collagen IV on day 7 and 12 (Figures 3 and 4). The morphological differences mentioned appeared consistently in all wells with non‐TAFs or TAFs respectively, and in the control wells.

### 3.3 Assessment of an invasive phenotype and migratory activity in the 3D DCIS co‐culture model

**FIGURE 5:**
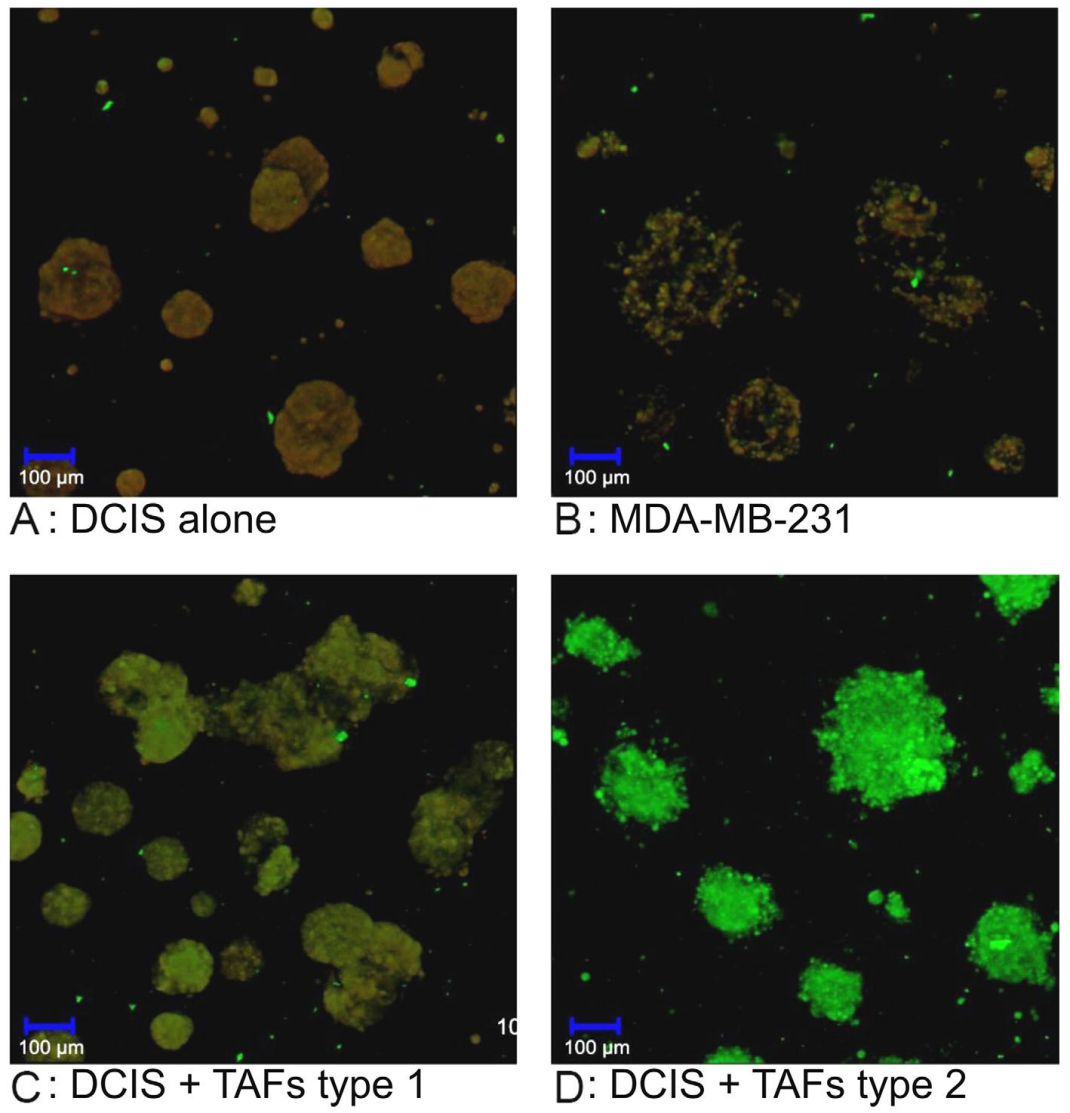
Assessment of the invasive phenotype by confocal microscopy. Spheroids of ductal carcinoma in situ (DCIS) cells, day 7 (A), MDA‐MB‐231 cells as an example of invasive spheroids, day 7 (B), and of DCIS cells in co‐culture with tumor associated fibroblasts (TAFs) of type 1, day 7 (C) or type 2, day14 (D) under the confocal microscope. The spheroids in (C) and (D) are morphologically similar to the spheroids in (B). Therefore, the morphological differences between the spheroids in (C) and (D) with the spheroids in A could correspond to the development of an invasive phenotype

The spheroid morphology of an invasive cell line was also examined to better assess the significance of the morphological differences. The triple‐negative, invasive breast cancer cell line MDA‐ΜΒ‐231 forms spheroids that are uneven and irregular in comparison to the MCF10DCIS.com cells in the control and similar to the MCF10DCIS.com cells in co‐culture with TAFs (Figure 5). We therefore conclude that the morphological differences in the spheroids co‐cultured with TAFs of type 1 and 2 correspond to the development of an invasive phenotype.

**FIGURE 6:**
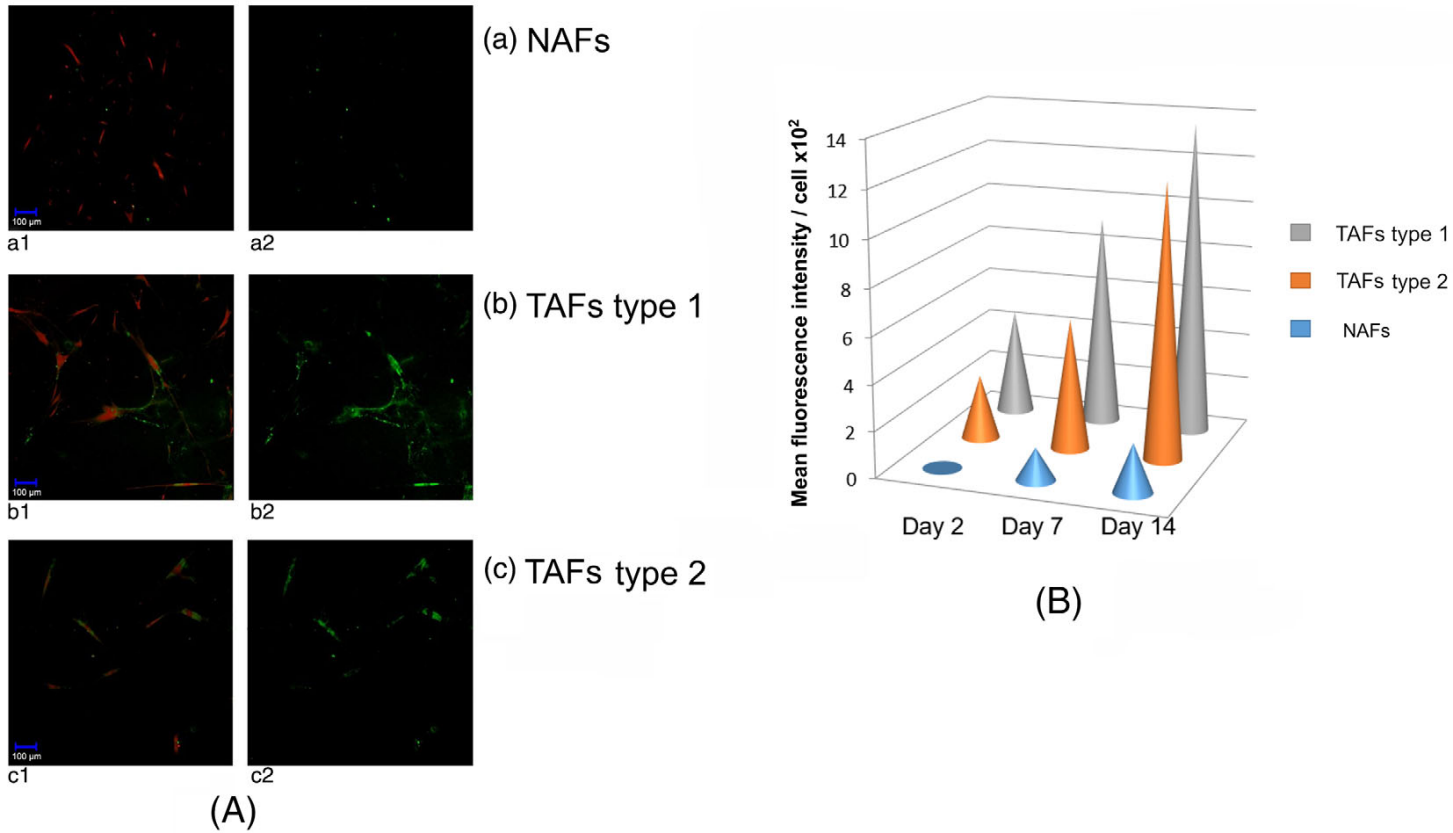
Analysis of the proteolytic activity of fibroblasts in the 3D co‐culture system by confocal microscopy. (A) Non‐tumor‐associated fibroblasts (NAFs) (a) and tumor‐associated fibroblasts (TAFs) of type 1 (b) and 2 (c) on day 2. Settings (magnification, laser intensity, gain for channel 1 and 2) are identical for the entire study. The TAFs have a stronger DQ‐collagen signal (b2, c2) than the NAFs (a2). The left panels show the cell tracker‐labeled cells using the two channels (HeNe543, argon diode 405‐30), while the green fluorescent signal isolated in the right panels labels degraded DQ‐collagen I. (B) Graphic: The mean fluorescence intensity per cell for NAFs (blue), TAFs of type 1 (orange) and 2 (gray) on day 2, 7, and 14. TAFs of type 2 show the greatest proteolytic activity. The laser intensity and gain for channel 2 was newly set on day 2, 7, and 14 to keep the signal‐to‐noise ratio as low as possible.

On day 2 as well as on day 7 and 14, a stronger intensity of the DQ‐collagen I signal of the TAFs of type 1 and 2 could be seen compared to the NAFs (Figure 6). The intensity of the DQ‐collagen I signal was quantified with the help of the ImageJ software. The mean fluorescence intensity per cell is shown in Figure 6. The TAFs showed a higher proteolytic activity compared to the NAFs on day 2 as well as on day 7 and 14.

**FIGURE 7:**
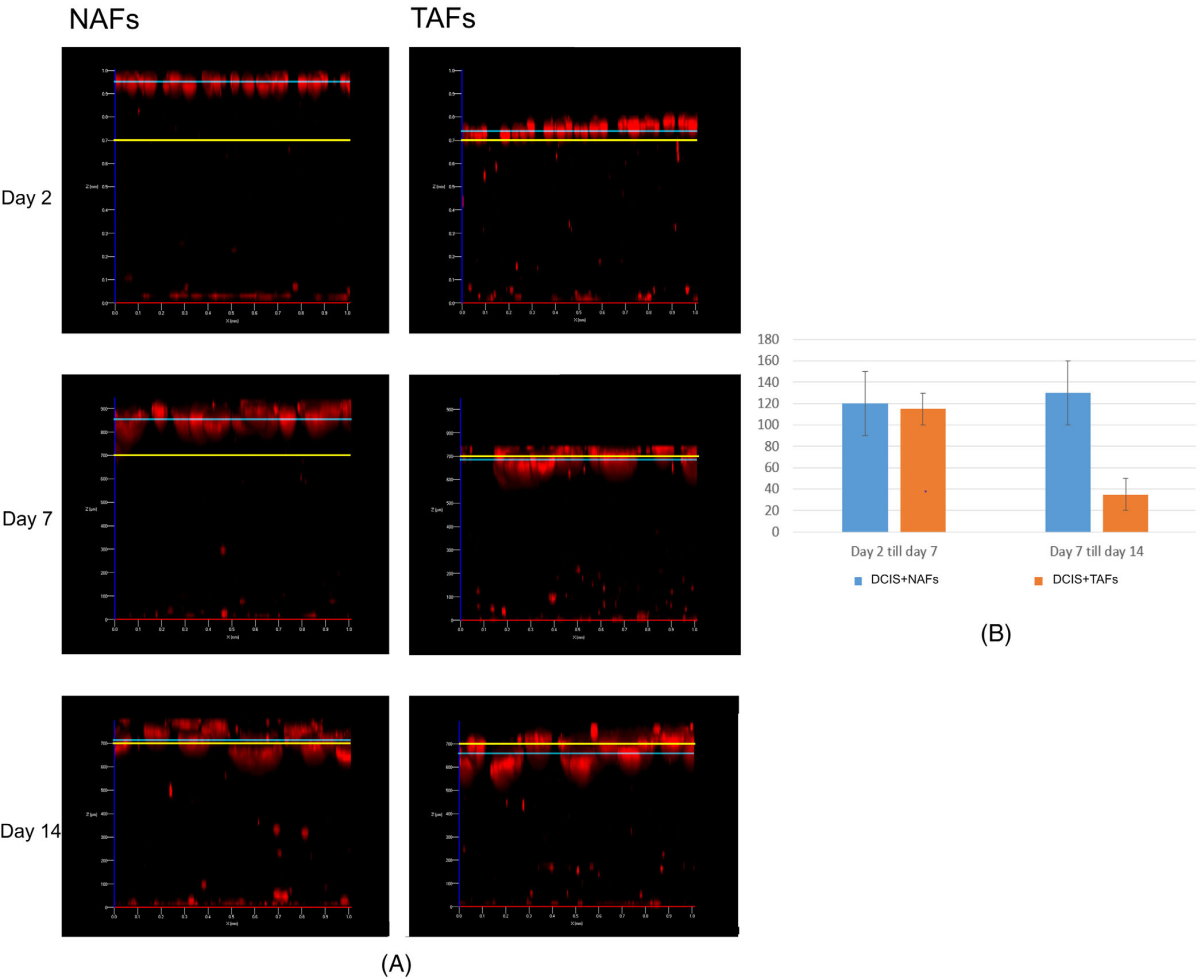
Assessment of cell movement in the 3D ductal carcinoma in situ (DCIS)‐ fibroblast co‐culture model. (A) Co‐culture of MCF10DCIS.com‐cells with non‐tumor‐associated fibroblasts (NAFs, left) and tumor‐associated fibroblasts (TAFs, right). The blue line marks the bottom of the spheroids. The yellow line marks a distance of 700 μm to the fibroblasts. The cells grow closer together over time, as the blue line crosses the yellow one. (B) Graphic: Average Distance Reduction with standard deviation between DCIS/NAFs (blue) and DCIS/TAFs (orange). There was no significant difference between the two groups (*p* = n.s)

Taken together the collagen and Matrigel layer were approximately 0.7–1 mm thick and so was the distance between fibroblasts and MCF10DCIS.com cells on day 2. On day 7 and 14 the distance became smaller. This applies to both TAFs and NAFs. The cells migrate towards each other as observed under the light microscope, too (Figures 1 and 7). In the experiment shown, the distance between MCF10DCIS.com cells and NAFs or TAFs is 890 μm and 700 μm on day 2, 760 μm and 560 μm on day 7 and 600 μm and 510 μm on day 14, respectively. This tendency has been observed in several experiments (n = 9). However, there was no significant difference in the mean distance reduction between NAFs and TAFs (day 2 to day 7: 120 ± 10 vs. 115 ± 15, *p* = .81, day 7 to 14: 130 ± 20 vs. 35 ± 15, *p* = .14) (Figure 7).

In summary, we show that MCF10DCIS.com cells form spheroids in Matrigel. The spheroid formation alone causes proteolysis indicated by the green DQ‐collagen signal. The co‐culture of MCF10DCIS.com cells with TAFs leads to the formation of irregular and uneven spheroids. This morphology corresponds to the morphology of the spheroids of invasive cells (MDA‐MB‐231). The TAFs also show an increased proteolytic activity compared to the NAFs, as evidenced by the stronger DQ‐collagen I signal. We additionally show that the cells apparently interact and migrate towards one another.

## 4 DISCUSSION

Compared to the other precursors, DCIS lesions have the highest risk of progressing to invasive breast cancer (15%–30%).^2^ The driving force behind this is not fully understood and clinical parameters to estimate the further course of the in situ lesion are lacking. Current research suggests that the TME plays a critical role in this transition.^17^, ^18^, ^19^, ^20^, ^21^, ^22^, ^23^, ^24^, ^25^ Further research into the causative factors for an invasion could enable personalized treatment. The aim of this work was to better characterize the role of fibroblasts in the conversion of DCIS to invasive breast cancer. For this purpose, based on the published MAME model,^27^ a 3D co‐culture model with fibroblasts primarily isolated from punch biopsies of the breast was established in order to initially look for morphological differences with confocal microscopy that indicate functional changes.

MCF10DCIS.com cells co‐cultured with NAFs form round and smooth spheroids in contrast to co‐culture with TAFs: here the spheroids are uneven and resemble the morphology of the spheroids of invasive cells, revealing the progression to an invasive phenotype of DCIS cells. The morphology of invasive spheroids with uneven surface, irregular shape and cells leaving the structure forming spikes has been described in the literature.^28^, ^29^, ^30^, ^31^, ^32^ As so, we interpret the morphological differences of the spheroids in co‐culture with TAFs as an indication that a reprogramming of fibroblasts into TAFs plays a role in the conversion of DCIS lesions to an invasive breast cancer. This coincides with the role of fibroblasts in the development of an in‐situ to an invasive phenotype in current data.^31^, ^32^, ^33^, ^34^, ^35^, ^36^

There is currently an intense debate on TAFs playing a crucial role in the development of DCIS into invasive breast cancer and that these could represent a new therapeutic target. The biology and physiology of the TAFs as well as the full activation mechanism is still subject of research. Various studies have described that TAFs can arise from several cell types; resident tissue fibroblasts, bone marrow‐derived mesenchymal stem cells, hematopoietic stem cells, epithelial cells (through epithelial‐mesenchymal transition/ EMT) or endothelial cells (through endothelial‐mesenchymal transition/ EndMT).^35^, ^36^

The aim of systemic therapy, for example, chemotherapy or endocrine therapy, is to destroy tumor cells. In most cases this is very successful in massively reducing the tumor mass. However, once the microenvironment has adopted a tumor‐promoting phenotype, restoration of tumor growth of a few cancer cells that may potentially escape first‐line therapy is very likely. Over 100 years ago, Paget et al. already suggested the importance of the TME with the “seed and soil” theory; the TME plays a critical role in the survival of tumor cells.^37^ Obviously, the microenvironment can lead to renewed tumor growth. The targeted treatment of the tumor‐promoting activities of TAFs is therefore an important therapeutic strategy.^38^

Primary human breast fibroblasts (HMF) stimulated with TGFβ1 have similar characteristics to breast cancer‐associated fibroblasts in vivo.^39^ It has also been proven that certain miRNAs promote the conversion of resident fibroblasts into TAFs.^40^ The elucidation of mechanisms that lead to the reprogramming of TAFs into normal fibroblasts could represent a new, promising therapeutic approach.^38^ From this perspective, experimental strategies like our 3D co‐culture system that address such issues are of high importance. Evaluating how current treatment strategies may affect cancer‐associated fibroblasts, or identifying effective drugs targeting these cancer‐associated cells may be the key in prohibiting or forecasting the progression of DCIS to invasive breast cancer.

The 3D mono‐culture of DCIS as well as 3D co‐culture models in which DCIS cells grow in an architecture resembling the cell: cell interactions or cell:ECM interactions are found in the literature (for comprehensive review see reference 41). These 3D approaches represent preclinical models either to examine different aspects of the tumormicroenvironment in the progression of DCIS to invasive breast cancer^33^, ^34^, ^42^ or to predict drug efficacy and toxicity.^43^, ^44^, ^45^ Studies on xenograft models have also been conducted to address the same questions^33^, ^46^, ^47^, ^48^, ^49^ (for comprehensive review see reference 50). 3D co‐culture models of TNBC (triple negative breast cancer) as well as xenograft models have also been used to recapitulate the breast tumor microenvironment and examine efficacy of therapeutic strategies.^51^

Τhe morphological differences between the spheroids presented in our work confirm that the TAFs are a critical entity in the development of an invasive lesion. The above‐mentioned invasive phenotype of the spheroids is associated with increased proteolysis in the TAFs. The fluorescence signal from DQ‐collagen I was stronger in the TAFs, which indicates the greater invasive potential of such cells compared to the NAFs. In addition, it was shown that co‐cultured MCF10DCIS.com cells and fibroblasts migrate towards one another (Figure 7). Sameni et al.^27^ have also observed that the distance between MCF10DCIS.com cells and TAFs (WS‐12Ti) becomes smaller over time. If more cells migrate or these cells migrate faster towards each other, when TAFs are involved still needs to be investigated.

Whereas 3D models to investigate DCIS are not new, our model provides novel insights. The innovation of our work compared to the MAME method published by Sameni et al.^27^ or other groups using culture models^33^ is that the co‐culture was partly carried out with primary cells and that we used fibroblasts of different biology (isolated from malignant or benign lesions) and not solely TAFs. This enables not only a better simulation of the in vivo situation compared to commercially available cell lines, but also a direct comparison between co‐culture of DCIS with NAFs or TAFs. Osuala et al did include NAFs and TAFs in their model to examine the IL‐6 signaling in the progression of DCIS to IBC. However, they examined neither differences in the morphology of the spheroids nor in the proteolytic activity between the co‐culture with NAFs or TAFs. Although primary cells better represent the tumor biology in vivo, their life span is limited compared to immortalized cell lines and genetic changes may occur with every cell cycle compared to stable, immortalized cells. Cell lines provide an infinite supply of a relatively homogeneous cell population, so it can be ensured that consecutive experiments have a largely identical cell population. A generalization of results with primary cells should be avoided.

Our work has some limitations. The experimental approaches/attempts are not completely identical because of the lack of influence on the number and size of the spheroids formed, although the experimental conditions were as identical as possible, and the number of cells seeded was kept the same. Under the general assumption that any experiment is never completely identical due to technical limitations, from our point of view the experiments carried out are well comparable with each other and allow not only morphological analysis, but also functional investigations planned in the future. Overall, our results regarding the morphology of the spheroids, the DQ‐collagen I signal and the distance between the cells were reproducible. In accordance to other research groups^27^, ^33^, ^34^ we evaluated proteolytic activity based on degraded DQ collagen signal. However, Yamada et. al showed that aggregated and/or mechanically disrupted collagen can also produce a DQ‐collagen type signal.^52^ Concequently, the use of controls with directly labeled matrix (e.g., Alexa‐labeled collagen) or with protease inhibitors could be used to rule out effects due to interactions with the local physical environment.

A weakness of in vitro cell cultures is that they are grown in the absence of their in vivo environment. In order to further investigate the development of an invasive breast cancer from a DCIS, other cell types, for example, cells of the immune system, could be included. The 3D‐culture is a design that better depicts the in‐vivo situation than 2D cultures. Nevertheless, results from the 3D‐culture should also be validated in vivo.

In summary, TAFs appear to play a crucial role in the development of DCIS lesions to invasive breast cancer. MCF10DCIS.com cells develop an invasive phenotype in co‐culture with TAFs, which have a greater proteolytic activity compared to NAFs. Our study provides the groundwork for follow‐up studies to further investigate the role of fibroblasts including the biological behavior of fibroblasts deriving from tumors with different tumor biology (e.g., luminal G3, Her2‐positive or triple‐negative carcinoma). In addition, we suggest that the morphological differences seen reflect genetic changes in proliferation and / or invasion markers. Our novel 3D‐culture model offers a simple in vitro model for the future study of molecular mechanisms underlying the communication of DCIS cells with their TME.

## AUTHOR CONTRIBUTIONS

**Marina Sourouni:** Conceptualization (lead); data curation (lead); formal analysis (lead); investigation (lead); methodology (lead); software (lead); visualization (lead); writing – original draft (lead); writing – review and editing (lead). **Carl Christian Opitz:** Resources (supporting); writing – original draft (supporting); writing – review and editing (supporting). **Isabel Radke:** Writing – original draft (supporting); writing – review and editing (supporting). **Ludwig Kiesel:** Supervision (supporting); writing – original draft (supporting); writing – review and editing (supporting). **Joke Tio:** Supervision (supporting); writing – original draft (supporting); writing – review and editing (supporting). **Martin Götte:** Methodology (supporting); supervision (supporting); writing – original draft (equal); writing – review and editing (equal). **Marie‐Kristin von Wahlde:** Conceptualization (equal); investigation (supporting); methodology (equal); supervision (equal); writing – original draft (supporting); writing – review and editing (equal).

## CONFLICT OF INTEREST

The authors have stated explicitly that there are no conflicts of interest in connection with this article.

## ETHICS STATEMENT

The study was carried out following the Declaration of Helsinki and approved by the local ethics commission (Ethikkommission der Ärztekammer Westfalen‐Lippe und der Medizinischen Fakultät der WWU; approval no.1 IX Greb 1 from 19 September 2001, updated 2012). The participants gave written informed consent.

## Data Availability

Not available.

## References

[1] Regerier AC , Possinger K . Mammakarzinom ‐ Manual Diagnostik und Therapie. Morphologische Grundlagen, Klassifikation, Stadieneinteilung; 2005 Available from: https://www.onkodin.de/e2/e32345/e32485/index_print_ger.html

[2] Hong YK , McMasters KM , Egger ME , Ajkay N . Ductal carcinoma in situ current trends, controversies, and review of literature. Am J Surg. 2018;216(5):998‐1003. 30244816 10.1016/j.amjsurg.2018.06.013

[3] Warnberg F , Garmo H , Emdin S , et al. Effect of radiotherapy after breast‐conserving surgery for ductal carcinoma in situ: 20 years follow‐up in the randomized SweDCIS trial. J Clin Oncol. 2014;32(32):3613‐3618. 25311220 10.1200/JCO.2014.56.2595

[4] Doke K , Butler S , Mitchell MP . Current therapeutic approaches to DCIS. J Mammary Gland Biol Neoplasia. 2018;23(4):279‐291. 30267199 10.1007/s10911-018-9415-1

[5] Stout NK , Cronin AM , Uno H , et al. Estrogen‐receptor status and risk of contralateral breast cancer following DCIS. Breast Cancer Res Treat. 2018;171(3):777‐781. 29946862 10.1007/s10549-018-4860-5

[6] Sprague BL , Trentham‐Dietz A , Nichols HB , Hampton JM , Newcomb PA . Change in lifestyle behaviors and medication use after a diagnosis of ductal carcinoma in situ. Breast Cancer Res Treat. 2010;124(2):487‐495. 20361251 10.1007/s10549-010-0869-0 PMC2924938

[7] Vatovec C , Erten MZ , Kolodinsky J , et al. Ductal carcinoma in situ: a brief review of treatment variation and impacts on patients and society. Crit Rev Eukaryot Gene Expr. 2014;24(4):281‐286. 25403959 10.1615/critreveukaryotgeneexpr.2014011495 PMC4372113

[8] Sourouni M . Hormonersatztherapie und duktales Carcinoma in situ. J für Gynäkologische Endokrinologie/ Schweiz. 2019;22(1):100‐104.

[9] Lazzeroni M , Dunn BK , Pruneri G , et al. Adjuvant therapy in patients with ductal carcinoma in situ of the breast: the Pandora's box. Cancer Treat Rev. 2017;55:1‐9. 28262606 10.1016/j.ctrv.2017.01.010

[10] O'Connell P , Pekkel V , Fuqua SA , Osborne CK , Clark GM , Allred DC . Analysis of loss of heterozygosity in 399 premalignant breast lesions at 15 genetic loci. J Natl Cancer Inst. 1998;90(9):697‐703. 9586667 10.1093/jnci/90.9.697

[11] Buerger H , Otterbach F , Simon R , et al. Comparative genomic hybridization of ductal carcinoma in situ of the breast‐evidence of multiple genetic pathways. J Pathol. 1999;187(4):396‐402. 10398097 10.1002/(SICI)1096-9896(199903)187:4<396::AID-PATH286>3.0.CO;2-L

[12] Buerger H , Otterbach F , Simon R , et al. Different genetic pathways in the evolution of invasive breast cancer are associated with distinct morphological subtypes. J Pathol. 1999;189(4):521‐526. 10629552 10.1002/(SICI)1096-9896(199912)189:4<521::AID-PATH472>3.0.CO;2-B

[13] Petridis C , Brook MN , Shah V , et al. Genetic predisposition to ductal carcinoma in situ of the breast. Breast Cancer Res. 2016;18(1):22. 26884359 10.1186/s13058-016-0675-7 PMC4756509

[14] Doebar SC , Sieuwerts AM , de Weerd V , Stoop H , Martens JWM , van Deurzen CHM . Gene expression differences between ductal carcinoma in situ with and without progression to invasive breast cancer. Am J Pathol. 2017;187(7):1648‐1655. 28634007 10.1016/j.ajpath.2017.03.012

[15] Hou L , Chen M , Wang M , et al. Systematic analyses of key genes and pathways in the development of invasive breast cancer. Gene. 2016;593(1):1‐12. 27506314 10.1016/j.gene.2016.08.007

[16] Schultz S , Bartsch H , Sotlar K , et al. Progression‐specific genes identified in microdissected formalin‐fixed and paraffin‐embedded tissue containing matched ductal carcinoma in situ and invasive ductal breast cancers. BMC Med Genomics. 2018;11(1):80. 30236106 10.1186/s12920-018-0403-5 PMC6147035

[17] Barsky SH , Karlin NJ . Myoepithelial cells: autocrine and paracrine suppressors of breast cancer progression. J Mammary Gland Biol Neoplasia. 2005;10(3):249‐260. 16807804 10.1007/s10911-005-9585-5

[18] Cowell CF , Weigelt B , Sakr RA , et al. Progression from ductal carcinoma in situ to invasive breast cancer: revisited. Mol Oncol. 2013;7(5):859‐869. 23890733 10.1016/j.molonc.2013.07.005 PMC5528459

[19] Sternlicht MD , Kedeshian P , Shao ZM , Safarians S , Barsky SH . The human myoepithelial cell is a natural tumor suppressor. Clin Cancer Res. 1997;3(11):1949‐1958. 9815584

[20] Barsky SH , Karlin NJ . Mechanisms of disease: breast tumor pathogenesis and the role of the myoepithelial cell. Nat Clin Pract Oncol. 2006;3(3):138‐151. 16520804 10.1038/ncponc0450

[21] Duivenvoorden HM , Rautela J , Edgington‐Mitchell LE , et al. Myoepithelial cell‐specific expression of stefin a as a suppressor of early breast cancer invasion. J Pathol. 2017;243(4):496‐509. 29086922 10.1002/path.4990

[22] Levental KR , Yu H , Kass L , et al. Matrix crosslinking forces tumor progression by enhancing integrin signaling. Cell. 2009;139(5):891‐906. 19931152 10.1016/j.cell.2009.10.027 PMC2788004

[23] Barker HE , Chang J , Cox TR , et al. LOXL2‐mediated matrix remodeling in metastasis and mammary gland involution. Cancer Res. 2011;71(5):1561‐1572. 21233336 10.1158/0008-5472.CAN-10-2868 PMC3842018

[24] Beguinot M , Dauplat MM , Kwiatkowski F , et al. Analysis of tumour‐infiltrating lymphocytes reveals two new biologically different subgroups of breast ductal carcinoma in situ. BMC Cancer. 2018;18(1):129. 29394917 10.1186/s12885-018-4013-6 PMC5797400

[25] Nelson AC , Machado HL , Schwertfeger KL . Breaking through to the other side: microenvironment contributions to DCIS initiation and progression. J Mammary Gland Biol Neoplasia. 2018;23(4):207‐221. 30168075 10.1007/s10911-018-9409-z PMC6237657

[26] Miller FR , Santner SJ , Tait L , Dawson PJ . MCF10DCIS.com xenograft model of human comedo ductal carcinoma in situ. J Natl Cancer Inst. 2000;92:1185‐1186. 10.1093/jnci/92.14.1185a 10904098

[27] Sameni M , Anbalagan A , Olive MB , Moin K , Mattingly RR , Sloane BF . MAME models for 4D live‐cell imaging of tumor: microenvironment interactions that impact malignant progression. J Vis Exp. 2012;60:3661. 10.3791/3661 PMC3376933 22371028

[28] Liu H , Lu T , Kremers GJ , Kremers GJ , Seynhaeve ALB , ten Hagen TLM . A microcarrier‐based spheroid 3D invasion assay to monitor dynamic cell movement in extracellular matrix. Biol Proc Online. 2020;22:3. 10.1186/s12575-019-0114-0 PMC6995242 32021568

[29] Lee GY , Kenny PA , Lee EH , Bissell MJ . Three‐dimensional culture models of normal and malignant breast epithelial cells. Nat Methods. 2007;4(4):359‐365. doi:10.1038/nmeth1015 17396127 PMC2933182

[30] Singh S , Tran S , Putman J , Tavana H . Three‐dimensional models of breast cancer‐fibroblasts interactions. Exp Biol Med (Maywood). 2020;245(10):879‐888. doi:10.1177/1535370220917366 32276543 PMC7268931

[31] Sameni M , Cavallo‐Medved D , Franco OE , et al. Pathomimetic avatars reveal divergent roles of microenvironment in invasive transition of ductal carcinoma in situ. Breast Cancer Res. 2017;19(1):56. 28506312 10.1186/s13058-017-0847-0 PMC5433063

[32] Olsen CJ , Moreira J , Lukanidin EM , Ambartsumian NS . Human mammary fibroblasts stimulate invasion of breast cancer cells in a three‐dimensional culture and increase stroma development in mouse xenografts. BMC Cancer. 2010;10:444. 20723242 10.1186/1471-2407-10-444 PMC2933628

[33] Jedeszko C , Victor BC , Podgorski I , Sloane BF . Fibroblast hepatocyte growth factor promotes invasion of human mammary ductal carcinoma in situ. Cancer Res. 2009;69(23):9148‐9155. 19920187 10.1158/0008-5472.CAN-09-1043 PMC2789178

[34] Osuala KO , Sameni M , Shah S , et al. Il‐6 signaling between ductal carcinoma in situ cells and carcinoma‐associated fibroblasts mediates tumor cell growth and migration. BMC Cancer. 2015;15:584. 26268945 10.1186/s12885-015-1576-3 PMC4535667

[35] Xing F , Saidou J , Watabe K . Cancer associated fibroblasts (CAFs) in tumor microenvironment. Front Biosci (Landmark Ed). 2010;15:166‐179. 20036813 10.2741/3613 PMC2905156

[36] Shiga K , Hara M , Nagasaki T , Sato T , Takahashi H , Takeyama H . Cancer‐associated fibroblasts: their characteristics and their roles in tumor growth. Cancers (Basel). 2015;7(4):2443‐2458. 26690480 10.3390/cancers7040902 PMC4695902

[37] Paget S . The distribution of secondary growths in cancer of the breast. 1889. Cancer Metastasis Rev. 1989;8(2):98‐101. 2673568

[38] Slany A , Bileck A , Muqaku B , Gerner C . Targeting breast cancer‐associated fibroblasts to improve anti‐cancer therapy. Breast. 2015;24(5):532‐538. 26210685 10.1016/j.breast.2015.06.009

[39] Groessl M , Slany A , Bileck A , et al. Proteome profiling of breast cancer biopsies reveals a wound healing signature of cancer‐associated fibroblasts. J Proteome Res. 2014;13(11):4773‐4782. 25238572 10.1021/pr500727h

[40] Mitra AK , Zillhardt M , Hua Y , et al. MicroRNAs reprogram normal fibroblasts into cancer‐associated fibroblasts in ovarian cancer. Cancer Discov. 2012;2(12):1100‐1108. 23171795 10.1158/2159-8290.CD-12-0206 PMC3685866

[41] Brock EJ , Ji K , Shah S , Mattingly RR , Sloane BF . In vitro models for studying invasive transitions of ductal carcinoma In situ. J Mammary Gland Biol Neoplasia. 2019;24(1):1‐15. doi:10.1007/s10911-018-9405-3 30056557 PMC6641861

[42] Rothberg JM , Sameni M , Moin K , Sloane BF . Live‐cell imaging of tumor proteolysis: impact of cellular and non‐cellular microenvironment. Biochim Biophys Acta. 2012;1824(1):123‐132. doi:10.1016/j.bbapap.2011.07.025 21854877 PMC3232330

[43] Farnie G , Johnson RL , Williams KE , Clarke RB , Bundred NJ . Lapatinib inhibits stem/progenitor proliferation in preclinical in vitro models of ductal carcinoma in situ (DCIS). Cell Cycle. 2014;13(3):418‐425. doi:10.4161/cc.27201 24247151 PMC3956537

[44] Williams KE , Bundred NJ , Landberg G , Clarke RB , Farnie G . Focal adhesion kinase and Wnt signaling regulate human ductal carcinoma in situ stem cell activity and response to radiotherapy. Stem Cells. 2015;33(2):327‐341. doi:10.1002/stem.1843 25187396

[45] Farnie G , Willan PM , Clarke RB , Bundred NJ . Combined inhibition of ErbB1/2 and notch receptors effectively targets breast ductal carcinoma in situ (DCIS) stem/progenitor cell activity regardless of ErbB2 status. PLoS One. 2013;8(2):e56840. doi:10.1371/journal.pone.0056840 23457626 PMC3572946

[46] Warnberg F et al. Effect of a farnesyl transferase inhibitor (R115777) on ductal carcinoma in situ of the breast in a human xenograft model and on breast and ovarian cancer cell growth in vitro and in vivo. Breast Cancer Res. 2006;8(2):R21. 16611371 10.1186/bcr1395 PMC1557711

[47] Spina V , Mariani BD , Gallagher RI , et al. Malignant precursor cells pre‐exist in human breast DCIS and require autophagy for survival. PLoS One. 2010;5(4):e10240. 20421921 10.1371/journal.pone.0010240 PMC2857649

[48] Behbod F , Kittrell FS , LaMarca H , et al. An intraductal human‐in‐mouse transplantation model mimics the subtypes of ductal carcinoma in situ. Breast Cancer Res. 2009;11(5):R66. 19735549 10.1186/bcr2358 PMC2790841

[49] Valdez KE , Fan F , Smith W , Allred DC , Medina D , Behbod F . Human primary ductal carcinoma in situ (DCIS) subtype‐specific pathology is preserved in a mouse intraductal (MIND) xenograft model. J Pathol. 2011;225(4):565‐573. 22025213 10.1002/path.2969 PMC3496769

[50] Behbod F , Gomes AM , Machado HL . Modeling human ductal carcinoma In situ in the mouse. J Mammary Gland Biol Neoplasia. 2018;23(4):269‐278. doi:10.1007/s10911-018-9408-0 30145750 PMC6244883

[51] Sameni M , Tovar EA , Essenburg CJ , et al. Cabozantinib (XL184) inhibits growth and invasion of preclinical TNBC models. Clin Cancer Res. 2016;22(4):923‐934. doi:10.1158/1078-0432.CCR-15-0187 26432786 PMC5500174

[52] Packard BZ , Artym VV , Komoriya A , Yamada KM . Direct visualization of protease activity on cells migrating in three‐dimensions. Matrix Biol. 2009;28(1):3‐10. doi:10.1016/j.matbio.2008.10.001 19010413 PMC2661756

